# An Instructive Case of Vomiting or Pregnancy

**Published:** 1884-11-20

**Authors:** John M. Upshur

**Affiliations:** Professor of Materia Medica and Therapeutics, Medical College of Virginia


### AN INSTRUCTIVE CASE OF VOMITING OF PREGNANCY.

By JOHN M. UPSHUR, M. D.,
Professor of Materia Medica and Therupeutics, Medical College of Virginia.

I was called on the 22d of March to see Mrs. ———, a primapara æt. 24, dark hair and eyes, fair complexion, and nervous temperament. Her last period occurred the latter part of January, so that at this time she was about the seventh week of pregnancy. Until now she had not suffered much with discomfort of any kind; but at the time I was applied to the nausea and vomiting were most distressing, waking her up at 3 a. m. every morning, and it being late in the day before she was even tolerably comfortable ; another paroxysm coming on in the evening, and lasting until nearly bed-time. Her tongue and general appearance indicated a torpid condition of the liver, so small doses (gr. $\frac{1}{4}$) of calomel were ordered every hour ; she was to take a milk-punch at bed-time, and a cup of strong coffee on awaking in the morning. The calomel produced three good bilious stools. The hips were elevated, and she was ordered to remain quietly in bed. Under this treatment she improved very much, but continued to have very distressing nausea in the morning, and her disgust for solid food exceeded anything of the kind I ever saw. She was now put on the much boasted ingluvien, and the result showed its utter worthlessness. On April 5 she had a well marked chill, followed by fever and a sweating stage, and the paroxysm accompanied by intolerable headache confined to the left fronto-parietal region. The attack lasted about six hours, and on subsiding left her comparatively comfortable. There was a recurrence on the second and third days. The question arose, Could it be malarial ? But she had not been exposed to malarial inflammation. Was it due to incipient uremia ? Analysis of the urine showed it to be normal. Was it, then, the beginning of septic poisoning, due to some morbid process in the womb, the death of the fetus from violent effort in vomiting, or some other cause of like nature ? The administration of grs. xviii of quinia sulph. successfully ended this complication ; but the morning nausea persisted, and the patient was becoming more prostrated, and her disgust to food increasing daily, obstinate hiccough set up. I had in the early treatment suspected displacement or some lesion of the womb as being an important factor in producing the nausea, but the patient's youth and natural delicacy made an examination per vagina very repulsive to her. But now things began to look serious, and I began to fear that her life would be the forfeit, either from exhaustion or hemorrhage from the stomach, unless she had speedy relief. I gave her per rectum 1 dr. of potass bromide, without effect, except that it produced agonizing pain. On April 13 I made a vaginal examination, and found the uterus enlarged to the size it should be at the tenth week of pregnancy, and retro-verted so that it lay flat upon the rectum its entire length, and evidently pressing on the sacral nerves. She had, a day or two previous to this examination, complained of severe pain in the back, especially when she assumed the dorsal decubitus. Here was an explanation of both pain and nausea. On the following day she was placed in the knee-chest position, and the womb easily replaced, and a Hodge's double lever (closed) pessary introduced to keep it in position. She had no more hiccough, and no more nausea until a week later, when she attempted to take a bath, and becoming very much exhausted, she fainted. She had been unable to go about her room since the introduction of the pessary ; was obliged now to keep her bed, and on the next morning she had an attack of nausea, but neither obstinate nor violent, followed by a severe nervous headache. On the next day she was able to be out of bed, and steadily improved until May 1. On the night before she imprudently had intercourse with her husband, attended with great sexual excitement on her part ; the next morning she felt nauseated, though she did not vomit ; felt badly all day, though being anxious to go to drive in the afternoon she said nothing of her uncomfortable feeling, but went to drive. She became nauseated while driving, fainted, and falling back the contents of her stomach were ejected either through her nose or retained in her mouth and throat, threatening suffocation. She would undoubtedly have died but that her husband, taking in the situation, changed her position. I saw her at 7.30 p. m. ; she was then much prostrated ; pulse 120 and thready ; stomach rejecting everything ; skin cool. Examination showed the pessary slightly misplaced ; womb below its proper position. Replaced pessary, and gave gtt. i of tincture of iodine in water about 11 p. m., when nausea ceased, and she has steadily improved until now (May 12), she is able to be out of bed and about her room ; appetite improving ; spirits good, and as she is advancing into the fourth month of pregnancy I fear no complication from nausea in the future.

### REMARKS.

The salient points in the above case are, first, the cause of the nausea primarily, namely, the retroverted womb misplaced by its increased weight in a state of pregnancy ; and, secondly, the recurrence of nausea under circumstances which produced nervous exhaustion when the system is enfeebled by conditions which had previously existed. The possible aggravation of morning sickness by the performance of the marriage relation makes it a question if it is not a thing to be forbidden in those cases which suffer from this symptom of pregnancy, certainly, until the uterus has risen out of the pelvis, and especially in those cases which are of a nervous, excitable temperament. The prompt relief from the drop-dose of iodine is also worthy of note, and for this suggestion I am indebted to Hewitt (Diseases of Women, vol 1, p. 398). As to the causes of this symptom we are much in the dark (Hewitt). The above author offers, as an explanation, flexion and versions of various kinds and degree, contending that the pinching of the nervous filaments on the concavity, and the stretching of the filaments of nerves on the convexity is the cause of this troublesome symptom. This may be so, though McClintock, Hewitt says, denies his position. Undoubtedly the above case proves that misplacement, so far as version goes, is a cause, or one of the causes. Tyler Smith says that pregnancy exerts an almost poisonous influence on some constitutions, and we have all seen vomiting a troublesome symptom when no displacement was to be found. Hewitt quotes many cases in his own practice in which this symptom was caused by displacement, and the causation is certainly sufficiently determined to make it incumbent upon us to carefully examine the womb in every case of obstinate vomiting. Ulceration or erosion of the cervix, or an indurated unyielding cervix may be causes, or the causes may be located in the body of the womb, simply the dilation of the womb under the growth of the fetus ; or there may be some diseased condition of the chorion, or of the placenta in its early formation. In my experience, a psychological cause may be the one at the root of the matter. I heard a brother M. D. say he had a patient who was violently nauseated even by the rattling of the dinner plates, and I had a case that could not bear the sight of a new spring dress without nausea ! To conclude the whole matter, we, of course, feel no apprehension, except in those cases in which life is endangered. Even mild cases may be aggravated to this point. So we cannot be too much on the alert, or too quick to discover the cause of the trouble in any and every case of vomiting in pregnancy; and I would earnestly urge the importance of keeping the patient from perturbing influences of all kinds ; and, in those cases where there is even a tendency to prostration, stimulation and a generous diet.—*American Journal of Obstetrics.*

